# Comparative Analysis of Bone Age Estimation Using the Manual Greulich and Pyle Method Versus Artificial Intelligence Algorithms in an Indian Pediatric Population Aged 1-18 Years

**DOI:** 10.7759/cureus.112210

**Published:** 2026-07-07

**Authors:** Prashant Onkar, Anushika Maloo, Suresh Phatak, Sandip Dhote, Kajal Mitra

**Affiliations:** 1 Department of Radiodiagnosis, NKP Salve Institute of Medical Science &amp; Research Centre and Lata Mangeshkar Hospital, Nagpur, IND; 2 Department of Radiodiagnosis, Datta Meghe Institute of Medical Sciences, Wardha, IND; 3 Department of Radiology, NKP Salve Institute of Medical Science &amp; Research Centre and Lata Mangeshkar Hospital, Nagpur, IND

**Keywords:** artificial intelligence, bone age assessment, greulich and pyle atlas, hand radiography, pediatric radiology, skeletal maturation

## Abstract

Introduction: Bone age (BA) estimation is an essential tool in pediatric growth assessment and endocrine evaluation. The study aimed to compare the performance and agreement of three artificial intelligence (AI)-based bone age assessment software platforms with the manual Greulich and Pyle (GP) atlas method in an Indian pediatric population.

Materials and methods: Seventy-four hand radiographs from children aged 1-18 years were analyzed retrospectively. BA was assessed using the manual GP atlas and three automated AI software platforms: Xrayhead (AI1), 16-Bit Physis (AI2), and FreeBoneAge (AI3). Statistical evaluation included Pearson correlation, Mean Absolute Error (MAE), Root Mean Square Error (RMSE), and Bland-Altman analysis to determine agreement and systematic bias between methods.

Results: All AI platforms showed significant correlation with the manual GP atlas (p < 0.001). Among the evaluated systems, 16-Bit Physis demonstrated the strongest agreement with GP assessment (ICC = 0.983, 95% CI: 0.973-0.989), the narrowest limits of agreement (3.53 years), and the lowest AI-related mean absolute error (1.24 years). FreeBoneAge showed good agreement (ICC = 0.903) but wider limits of agreement, whereas Xrayhead demonstrated only moderate agreement (ICC = 0.729) and produced unreliable outputs in children younger than six years. The manual GP method achieved the lowest overall mean absolute error relative to chronological age (0.76 years).

Conclusion: 16-Bit Physis AI demonstrated the most reliable agreement with the GP atlas across all age groups and both sexes, with clinically acceptable bias and narrow limits of agreement. Xrayhead showed critical limitations in young children.

## Introduction

Bone age assessment is an important tool in pediatric growth evaluation and is widely used in the diagnosis of endocrine disorders, growth abnormalities, and pubertal disturbances [1]. The Greulich and Pyle (GP) atlas remains the most commonly used method because of its simplicity and widespread clinical acceptance [2]. However, manual assessment is time-consuming and subject to interobserver variability [2].

Recent advances in artificial intelligence (AI) have led to the development of automated bone age assessment software platforms aimed at improving reproducibility and reducing reporting time. Previous studies have demonstrated strong agreement between AI-based methods and the conventional GP method. However, significant variability has been reported among different AI platforms, emphasizing the need for independent validation before clinical implementation [3].

Furthermore, most validation studies have been performed in Western populations, and data regarding the performance of these systems in Indian children remain limited. Therefore, this study aimed to compare bone age estimation obtained using three AI-based platforms: Xrayhead [4], 16-Bit Physis [5], and FreeBoneAge [6] with the conventional manual GP method in Indian children aged 1-18 years.

## Materials and methods

Study design

This retrospective study was conducted in the Department of Radiodiagnosis, NKP Salve Institute of Medical Science & Research Centre and Lata Mangeshkar Hospital (NKPSIMS & RC and LMH), where hand radiographs were acquired over six months (from March 2025 to August 2025). Image analysis and statistical evaluation were subsequently performed after completion of data collection. Institutional Ethics Committee (LMH/IEC/5/2025, dated January 30, 2025) approval was obtained prior to the commencement of the study. All radiographs and demographic data were anonymized before analysis, and patient confidentiality was maintained throughout the study.

Study population and sampling

The study included the Indian pediatric population who underwent hand and wrist radiography for routine clinical indications at our institution. Radiographs obtained for patients with skeletal dysplasia, endocrine disorders affecting skeletal maturation, metabolic bone disease, fractures, or poor image quality were excluded. The total sample size was 74 participants, with 48 males (64.9%) and 26 females (35.1%) (mean chronological age 11.54 ± 4.93 years; range 1.0-18.0 years).

Inclusion Criteria

Children aged 1-18 years who underwent left hand and wrist radiography during the study period and had radiographs of adequate diagnostic quality were included.

Exclusion Criteria

Radiographs of children with skeletal dysplasia, metabolic bone disease, endocrine disorders known to affect skeletal maturation, previous hand or wrist fractures, orthopedic hardware involving the imaged hand or wrist, or poor image quality were excluded.

Method of measurement

**Figure 1:**
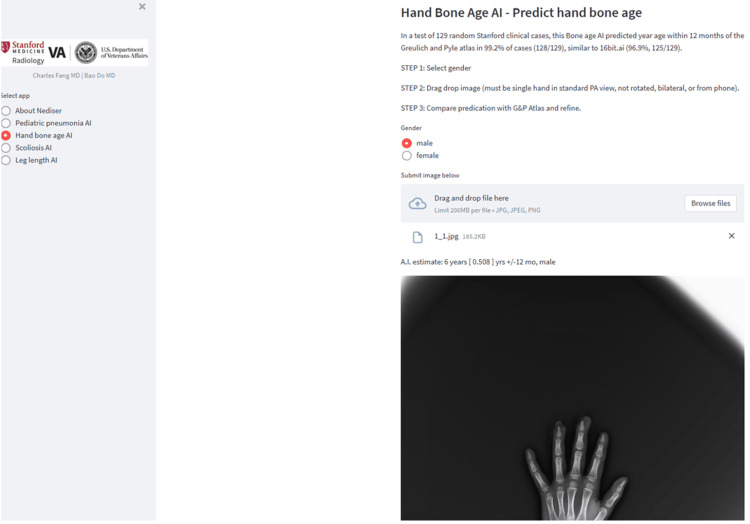
Automated bone age assessment output generated by the Xrayhead platform. The figure demonstrates the Xrayhead software interface displaying automated skeletal maturity assessment from a left hand radiograph of a five-year-old child. The estimated bone age generated by Xrayhead for this representative case was six years, as displayed on the software interface.

**Figure 2:**
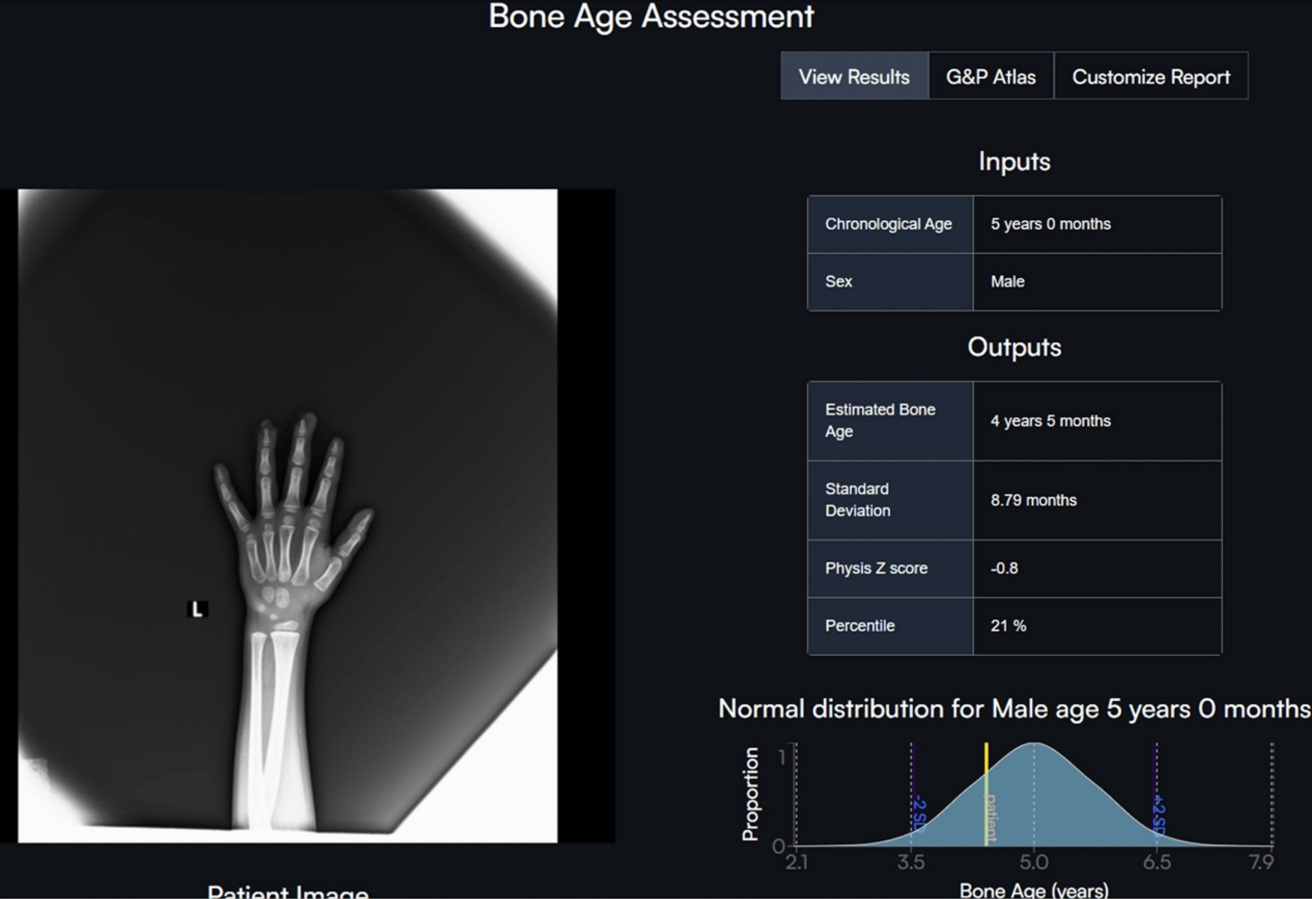
Automated bone age assessment output generated by the 16-Bit Physis platform. The figure demonstrates the 16-Bit Physis software interface displaying automated bone age estimation from the same left hand radiograph of a five-year-old child. The platform provides skeletal maturity assessment and the corresponding bone age estimate derived using its artificial intelligence-based algorithm.

**Figure 3:**
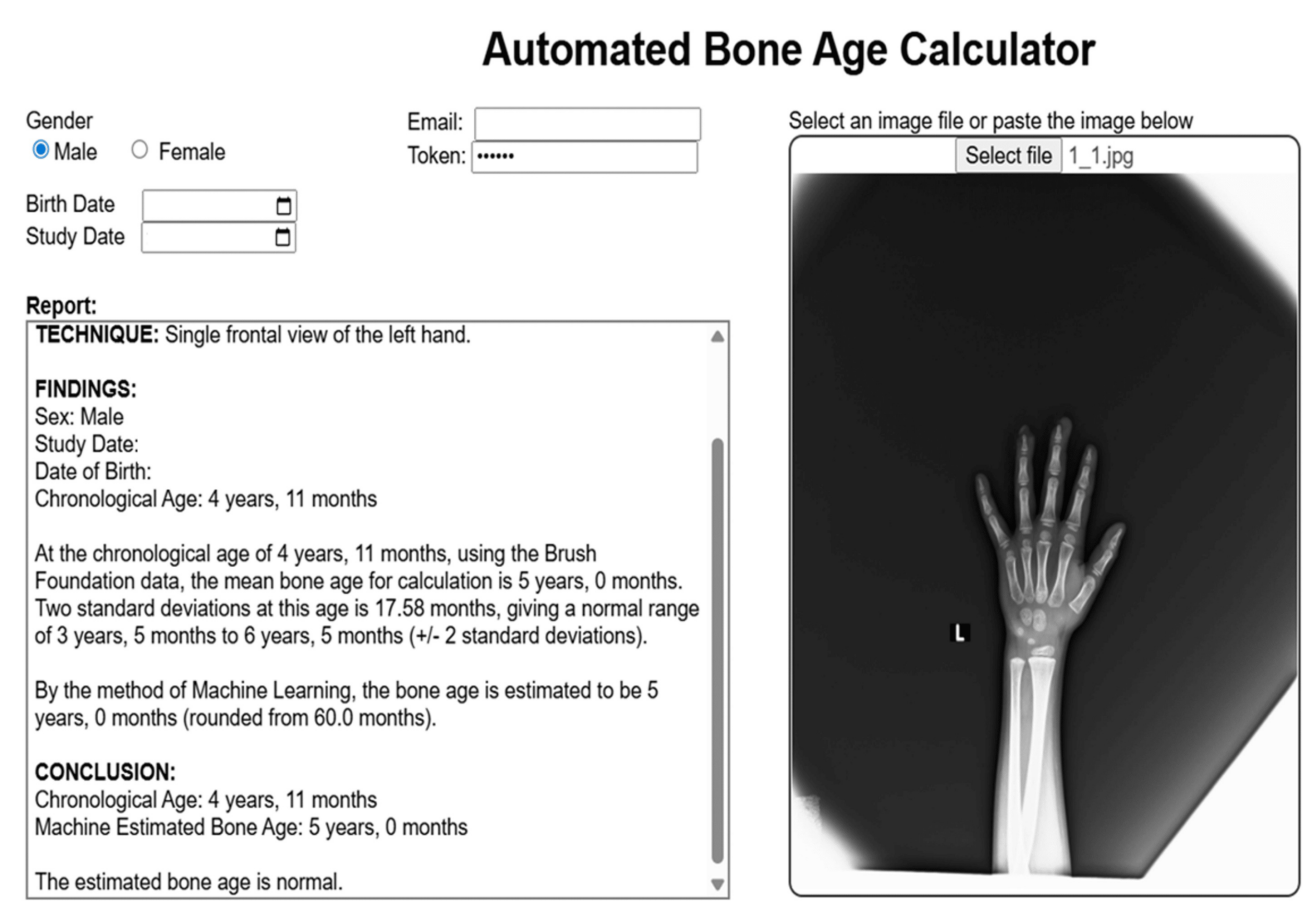
Automated bone age assessment output generated by the FreeBoneAge platform. The figure demonstrates the FreeBoneAge software interface displaying automated bone age estimation from the same left-hand radiograph of a five-year-old child. The estimated skeletal age generated by the algorithm is shown together with the associated image analysis output used for bone age assessment.

Bone age was estimated for all participants satisfying the criteria. Manual GP bone age assessment was performed by a radiologist with a few years of experience using the GP atlas. The same radiographs were then processed through three AI software platforms as shown in Figures 1-3. AI-based analysis was performed independently after image anonymization. The software platforms generated automated bone age estimates without access to the manual GP assessment results. The radiologist performing manual GP assessment was blinded to the AI-generated outputs during interpretation. Bone age estimates from all methods were noted and compared against the chronological age of the subjects. Chronological age was used only as a comparative reference and not as the reference standard for skeletal maturity. The AI software platforms used were: (i) Xrayhead (Stanford University; web-based platform) (AI1); (ii) 16-Bit Physis (AI2); (iii) FreeBoneAge (AI3).

Based on platform behavior observed during analysis, Xrayhead returned a minimum estimated bone age of six years for younger children and was therefore considered unreliable for participants younger than six years.

All measurements were entered into a standardized Microsoft Excel (Microsoft Corporation, Redmond, WA, USA) spreadsheet by the primary investigator. Data entries were cross-checked against the original records prior to statistical analysis to minimize transcription errors.

Statistical analysis

Agreement between each AI software platform and the GP atlas was assessed by Bland-Altman analysis (bias = mean GP minus AI; 95% limits of agreement (LoA) = bias ± 1.96 × SD of differences). Because the study variables (chronological age, GP atlas, and all three AI platforms) were non-normally distributed, Spearman rank (ρ) correlation was considered the primary measure of association. Pearson correlation coefficients (r) are presented as supplementary descriptive measures, whereas agreement was primarily assessed using the Intraclass Correlation Coefficient and Bland-Altman analysis. Intraclass Correlation Coefficient (ICC(3,1), two-way mixed effects model, absolute agreement, single measures) was additionally calculated for each AI platform versus the GP atlas to quantify method agreement beyond linear association. A two-way mixed-effects ICC model for absolute agreement (ICC(3,1)) was selected because the same fixed measurement methods (manual GP atlas and the respective AI platform) were applied to all study participants. N and % values are presented together throughout. The difference from chronological age was measured by Mean Absolute Error (MAE) and Root Mean Square Error (RMSE). Subgroup analyses were performed by age group (1-6, 7-12, 13-18 years) and sex. Continuous variables were summarized using median values and mean ± standard deviation for descriptive comparison. Because variables were non-normally distributed on Shapiro-Wilk testing, Spearman rank correlation was used as the primary measure of association. Statistical significance threshold: p < 0.05. Raw data for all cases are presented in Table 1.

**Table 1:** Raw dataset of study participants including chronological age, manual Greulich and Pyle (GP) bone age, and automated bone age estimates. The table presents chronological age (CA), manual GP atlas bone age, and automated bone age estimates generated by Xrayhead (AI1), 16-Bit Physis (AI2), and FreeBoneAge (AI3) for all 74 participants. Sex is presented as male (M) or female (F). CA = chronological age; GP = Greulich and Pyle atlas; AI1 = Xrayhead; AI2 = 16-Bit Physis; AI3 = FreeBoneAge.

Case No.	Gender	CA (yrs)	GP atlas (yrs)	Xrayhead AI1 (yrs)	16-Bit Physis AI2 (yrs)	FreeBoneAge AI3 (yrs)
1	M	3.00	4.00	6.70	4.08	3.50
2	M	6.00	8.00	6.50	7.92	6.00
3	F	13.00	12.00	13.55	12.25	11.00
4	F	4.00	5.00	6.34	4.83	4.17
5	M	4.00	4.00	6.91	4.67	4.50
6	F	9.08	7.83	14.25	7.75	5.75
7	F	2.00	3.50	14.40	3.75	4.16
8	M	18.00	17.00	16.54	16.58	19.00
9	M	13.00	14.00	12.39	14.33	13.00
10	M	5.00	7.00	6.54	6.58	5.00
11	F	2.00	1.50	6.26	1.67	2.50
12	F	3.00	2.50	16.29	2.33	7.83
13	F	16.00	14.00	16.25	14.25	12.00
14	F	16.00	15.00	16.55	14.83	16.00
15	F	12.00	13.00	15.37	12.67	12.00
16	M	17.00	17.00	16.47	16.75	15.00
17	M	6.17	8.00	6.54	7.92	6.00
18	M	17.00	16.00	16.56	16.25	18.00
19	F	16.00	12.00	16.48	12.25	5.00
20	M	16.00	16.00	16.46	16.33	17.00
21	M	10.00	9.00	6.64	8.91	6.00
22	M	11.00	11.50	6.14	11.42	11.50
23	F	15.00	15.00	13.39	15.42	14.00
24	F	10.00	14.00	13.48	14.33	13.50
25	F	14.00	15.00	13.00	14.90	11.00
26	M	17.00	17.50	15.00	17.80	18.00
27	F	18.00	16.00	14.00	14.44	19.00
28	M	16.00	16.00	16.00	16.62	14.44
29	M	6.00	6.00	6.00	9.90	6.33
30	M	16.00	16.00	16.50	16.90	18.00
31	M	10.00	9.00	6.71	7.50	11.00
32	M	6.00	6.00	6.40	5.40	8.00
33	M	5.00	5.00	6.47	4.80	5.50
34	M	2.00	2.00	6.00	1.20	6.00
35	F	17.00	17.00	13.30	15.11	15.00
36	F	16.00	15.00	13.00	12.40	16.00
37	M	16.00	16.00	16.70	16.00	16.33
38	M	17.00	17.00	16.30	16.80	19.00
39	M	15.00	15.00	13.29	15.11	16.00
40	M	10.00	10.00	6.19	9.80	11.33
41	F	4.00	4.00	14.00	2.50	3.00
42	M	5.00	5.00	6.50	4.50	4.00
43	F	17.00	17.00	14.30	15.20	17.00
44	F	17.00	17.00	14.50	15.40	19.00
45	F	10.00	10.00	13.40	11.60	8.66
46	M	12.00	12.00	9.00	12.40	13.80
47	F	17.00	17.00	14.50	15.90	13.66
48	M	17.00	17.00	16.00	16.90	16.00
49	M	17.00	17.00	16.00	16.40	17.44
50	F	12.00	12.00	14.00	10.30	13.00
51	F	4.00	4.00	6.00	2.00	5.00
52	M	14.00	17.00	16.00	17.40	14.66
53	M	2.00	2.00	6.76	1.60	5.00
54	M	13.00	15.00	14.00	15.90	16.00
55	M	12.00	13.00	12.30	13.50	12.66
56	M	12.00	14.00	12.19	13.70	12.55
57	F	13.00	13.00	14.48	13.40	15.00
58	M	13.00	14.00	14.53	14.20	16.00
59	M	14.00	14.00	12.28	14.40	15.00
60	M	10.00	11.50	6.14	11.00	11.00
61	M	14.00	15.00	15.40	15.00	13.00
62	M	17.00	17.00	16.00	16.80	18.30
63	M	11.00	12.60	12.15	12.30	11.50
64	M	14.00	15.50	14.50	15.60	18.00
65	M	6.00	6.00	6.90	6.40	8.00
66	M	14.00	15.00	14.31	15.20	16.00
67	F	14.00	14.00	13.43	14.30	12.00
68	M	17.00	17.00	16.00	17.50	17.00
69	M	8.00	10.00	11.00	10.00	9.00
70	F	12.00	12.00	13.50	12.10	14.00
71	M	14.00	14.00	13.00	15.60	17.00
72	M	13.00	14.00	13.61	13.80	11.00
73	M	14.00	17.00	16.58	17.50	11.00
74	M	6.00	6.00	6.86	5.90	9.00

## Results

Study demographics and descriptive statistics

Seventy-four children were included (48 male, 64.9%; 26 female, 35.1%). Age distribution: 1-6 years (n = 18, 24.3%), 7-12 years (n = 17, 23.0%), 13-18 years (n = 39, 52.7%). Descriptive statistics are presented in Table 2. Although the subgroup was categorized as 1-6 years to reflect clinically relevant pediatric age bands, the youngest participant included in this study was 2 years of age.

**Table 2:** Descriptive statistics of chronological age and bone age estimates obtained by different assessment methods. The table summarizes chronological age and bone age estimates obtained using the manual Greulich and Pyle (GP) atlas and the three automated bone age assessment platforms. Continuous variables are presented as mean ± standard deviation (SD), median, minimum, and maximum values in years. Study population characteristics are presented as n (%). A p-value <0.05 was considered statistically significant. Xrayhead minimum output of 6.0 years reflects its lower age limit (actual study minimum 2.0 years chronologically). SD = standard deviation.

Statistic	Chronological age	GP atlas	Xrayhead (AI1)	16-Bit Physis (AI2)	FreeBoneAge (AI3)
Mean (SD), years	11.54 (4.93)	11.84 (4.82)	12.19 (3.91)	11.74 (4.89)	11.85 (4.87)
Median, years	13.00	14.00	13.49	13.60	12.61
Range (min-max), years	2.0-18.0	1.5-17.5	6.0-16.7	1.2-17.8	2.5-19.0

Correlation and agreement analysis (Spearman, Pearson, and ICC)

All AI software platforms demonstrated positive associations with both manual GP and chronological age; however, the primary findings were the differences in agreement, ICC values, MAE/RMSE, and Bland-Altman limits of agreement between platforms (Pearson and Spearman tests; Table 3). The 16-Bit Physis AI software platform demonstrated the strongest agreement with the GP atlas (ICC = 0.983; LoA range 3.53 years), followed by FreeBoneAge (ICC = 0.903; LoA range 8.44 years), whereas Xrayhead showed the weakest agreement (ICC = 0.729; LoA range 12.66 years).

**Table 3:** Correlation and agreement analysis between chronological age, manual Greulich and Pyle atlas assessment, and automated bone age assessment platforms. The table presents Pearson correlation coefficients (r), Spearman rank correlation coefficients (ρ), corresponding test statistics, and Intraclass Correlation Coefficients (ICC(3,1)) with 95% confidence intervals for comparisons between chronological age, manual GP assessment, and automated bone age estimates. Correlation coefficients and agreement statistics are presented as numerical values with corresponding 95% confidence intervals where applicable. Statistical significance was defined as p <0.05. CA = chronological age; GP = Greulich and Pyle atlas; ICC = Intraclass Correlation Coefficient; CI = confidence interval.

Method	Pearson r vs. CA	Spearman ρ vs. CA	Spearman t (CA)	Pearson r vs. GP	Spearman ρ vs. GP	Spearman t (GP)	ICC(3,1) (95% CI)
GP atlas (manual)	0.970	0.951	26.03	1.000	1.000	N/A	N/A (reference)
Xrayhead (AI1)	0.757	0.718	8.76	0.745	0.711	8.58	0.729 (0.602-0.820)
16-Bit Physis (AI2)	0.947	0.905	18.00	0.983	0.969	33.31	0.983 (0.973-0.989)
FreeBoneAge (AI3)	0.890	0.872	15.09	0.901	0.878	15.58	0.903 (0.848-0.937)

Bland-Altman agreement analysis

**Figure 4:**
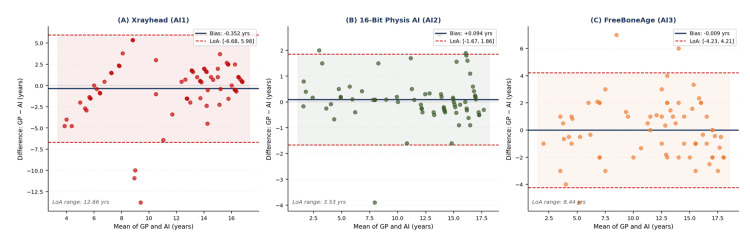
Bland–Altman plots demonstrating agreement between the manual Greulich and Pyle (GP) method and AI-based bone age assessment platforms. (A) GP versus Xrayhead; (B) GP versus 16-Bit Physis; (C) GP versus FreeBoneAge. The solid horizontal line represents the mean bias (GP-AI estimate), while the dashed lines indicate the 95% limits of agreement (mean bias ± 1.96 SD). The x-axis represents the mean bone age of the GP and AI methods (years), and the y-axis represents the difference between the GP and AI estimates (years). Narrower limits of agreement indicate closer agreement with the manual GP method. GP = Greulich and Pyle; AI = artificial intelligence; SD = standard deviation.

Table 4 and Figure 4 present Bland-Altman results. The 16-Bit Physis AI software platform demonstrated the narrowest limits of agreement (LoA range 3.53 years; bias +0.094 years), indicating relatively close agreement with the manual GP reference method. FreeBoneAge showed near-zero bias (-0.009 years) but wider LoA (range 8.44 years). Xrayhead had the widest LoA (range 12.66 years) and a negative bias of -0.352 years, reflecting systematic overestimation and high individual variability. 

**Table 4:** Bland-Altman agreement analysis (bias = GP minus AI). Data are presented as mean ± SD, median, or n (%), as appropriate. A p-value < 0.05 was considered statistically significant. Bias = mean difference (GP minus AI method). Positive bias: AI reads lower than GP; Negative bias: AI reads higher than GP. GP = Greulich and Pyle atlas; AI = artificial intelligence; SD = standard deviation; LoA = 95% Limits of Agreement (bias ± 1.96×SD). N = 74. Statistical significance threshold: p < 0.05. Bland-Altman difference scores were non-normally distributed for all three comparisons (Shapiro-Wilk: GP−AI1 W=0.857, p=6.33×10⁻⁷; GP−AI2 W=0.870, p=1.83×10⁻⁶; GP−AI3 W=0.967, p=0.049).

Comparison	Bias (years)	SD (years)	LoA lower	LoA upper	LoA range
GP vs. Xrayhead	-0.352	3.230	-6.683	+5.979	12.66
GP vs. 16-Bit Physis	+0.094	0.905	-1.668	+1.857	3.53
GP vs. FreeBoneAge	-0.009	2.161	-4.226	+4.209	8.44

Because the Bland-Altman difference distributions deviated from normality, these analyses were interpreted as exploratory agreement assessments. The Bland-Altman plots (Figure 4) were reviewed for evidence of skewness, funneling, or increasing spread at higher mean values, and no major pattern suggesting substantial heteroscedasticity was observed.

Accuracy: MAE and RMSE vs. chronological age

**Figure 5:**
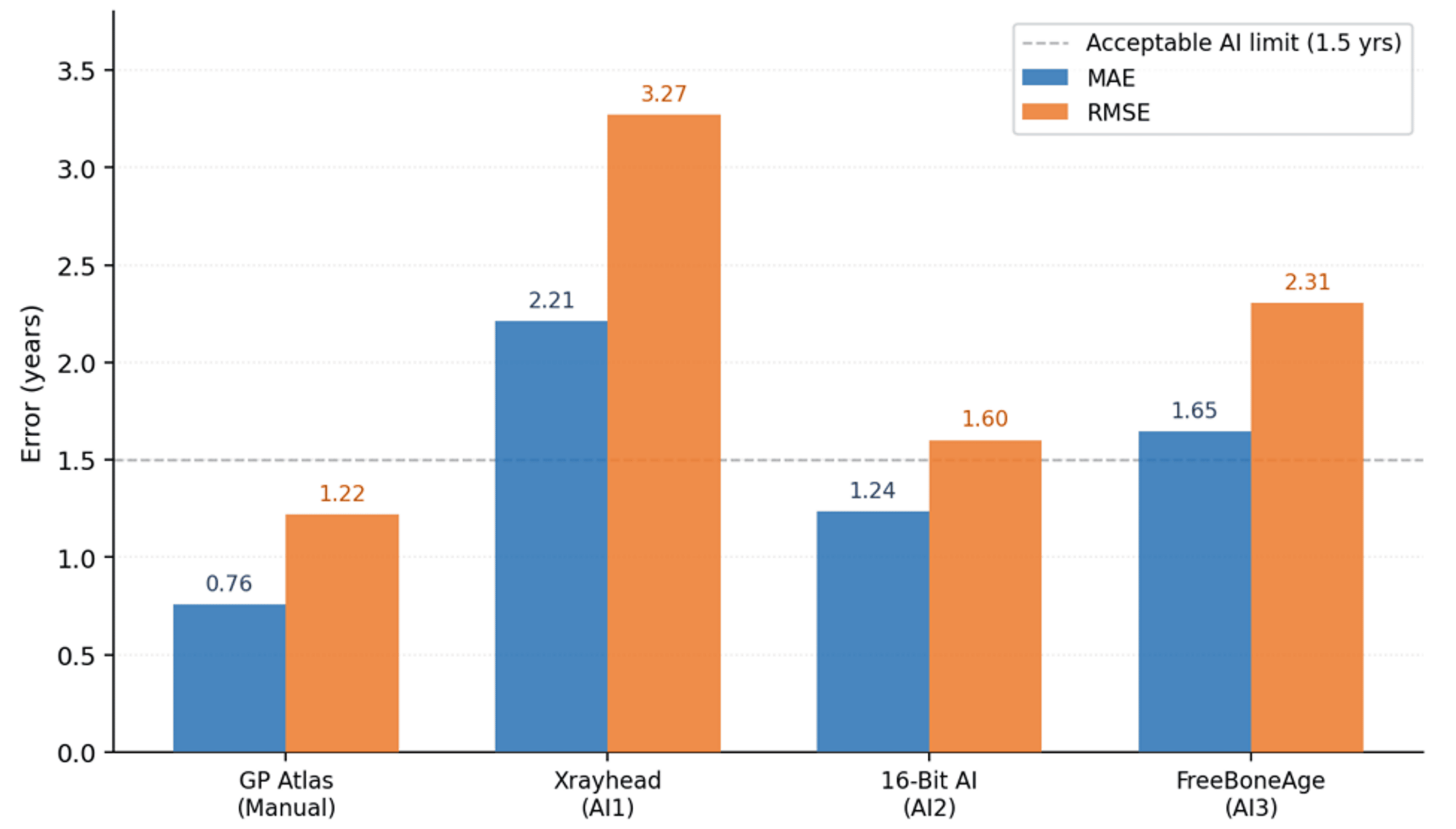
Comparison of accuracy metrics for manual and automated bone age assessment methods relative to chronological age. The graph demonstrates Mean Absolute Error (MAE) and Root Mean Square Error (RMSE) for the Greulich and Pyle (GP) atlas, Xrayhead (AI1), 16-Bit Physis (AI2), and FreeBoneAge (AI3) relative to chronological age. Lower values indicate greater agreement and the smallest difference from chronological age among the evaluated AI platforms. The 16-Bit Physis AI software platform demonstrated the lowest MAE and RMSE values, indicating the highest overall accuracy. The dashed horizontal line represents the approximate 1.5-year threshold considered clinically acceptable for automated bone age estimation. All values are presented in years.

The GP atlas achieved the lowest MAE (0.76 years; RMSE 1.22 years). Among AI software platforms, 16-Bit Physis demonstrated the lowest AI-related MAE (1.24 years; RMSE 1.60 years), suggesting relatively good agreement with chronological age, followed by FreeBoneAge (MAE 1.65 years) and Xrayhead (MAE 2.21 years; Table 5, Figure 5).

**Table 5:** MAE and RMSE for each method relative to chronological age. Data is presented in years. The dashed line marks the 1.5-year approximate threshold for clinically acceptable AI bone age error. Data are presented as mean ± SD, median, or n (%), as appropriate. A p-value < 0.05 was considered statistically significant. The dashed horizontal line at 1.5 years represents an exploratory benchmark included for visual comparison of method performance. Although no universally accepted clinical threshold exists for automated bone age assessment, previous validation studies have reported mean absolute errors of approximately 0.6-1.3 years for well-performing AI-based systems, placing values near 1.5 years at the upper end of reported performance rather than representing a validated clinical cutoff [3,7-9]. All values in years relative to chronological age. Dashed threshold line = 1.5 years (approximate published threshold for clinically acceptable AI bone age error). Xrayhead outputs a floor value of 6.0 years for children under 6 years (n=18, 24.3%), producing unreliable estimates in this age group. N = 74. MAE = Mean Absolute Error; RMSE = Root Mean Square Error; GP: Greulich and Pyle atlas.

Method	MAE (years)	RMSE (years)	Notes
GP atlas (manual)	0.76	1.22	Reference method
16-Bit Physis	1.24	1.60	Best AI - within acceptable range
FreeBoneAge	1.646	2.31	-
Xrayhead	2.210	3.27	≥6 years only; poorest accuracy

Subgroup analysis

**Figure 6:**
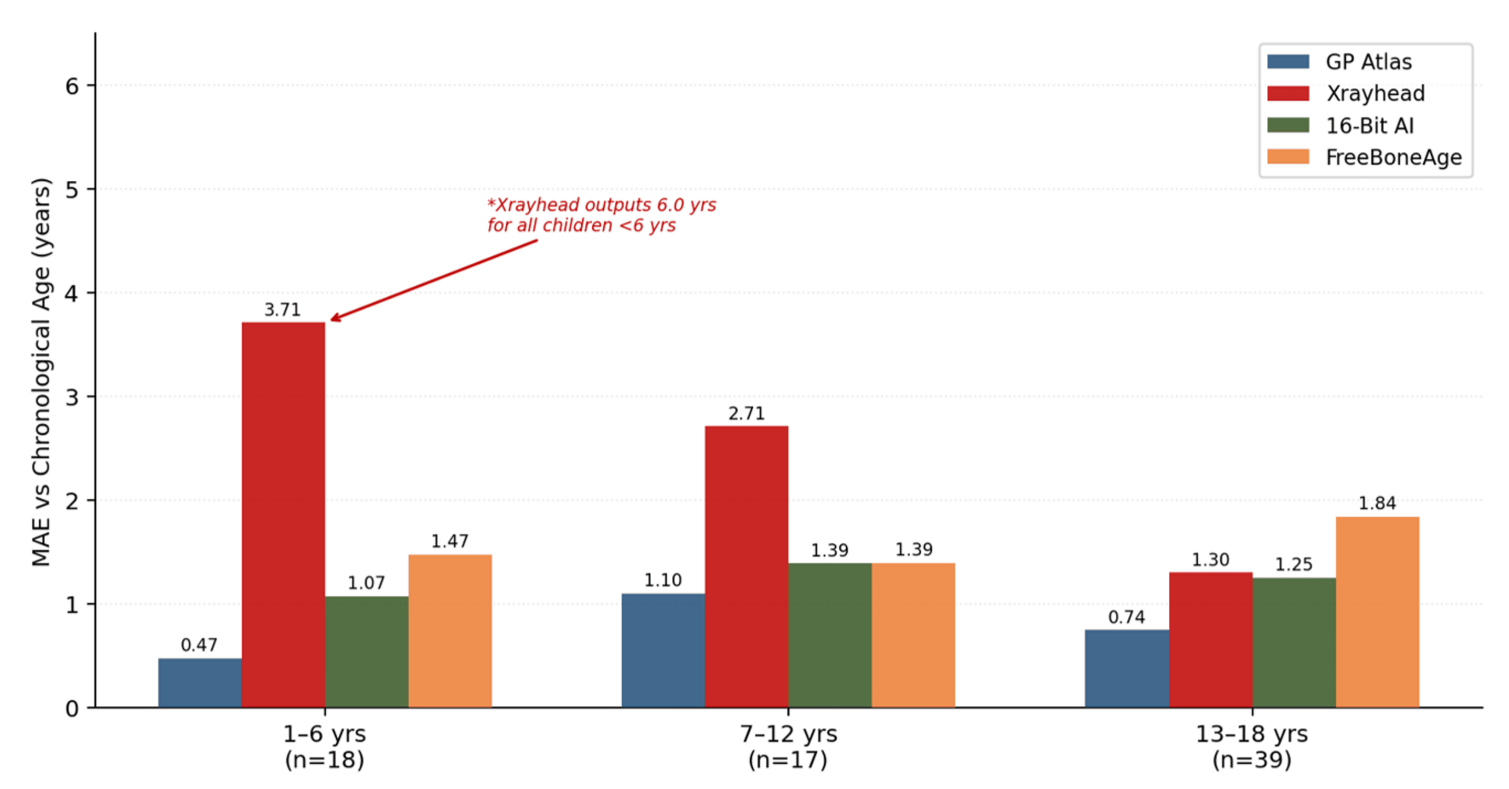
Mean Absolute Error (MAE) stratified by age group for each bone age assessment method. The graph compares MAE values across three age groups (1-6 years, 7-12 years, and 13-18 years) for the Greulich and Pyle (GP) atlas, Xrayhead, 16-Bit Physis, and FreeBoneAge. Lower MAE values indicate greater agreement with chronological age. The 16-Bit Physis AI software platform maintained relatively consistent performance across all age groups. Xrayhead demonstrated markedly increased error in the 1-6-year age group because the software generated a fixed minimum output of 6.0 years irrespective of actual skeletal maturity, resulting in clinically unreliable estimates in younger children. All values are presented in years.

Table 6 and Figure 6 show MAE by age group. Xrayhead produced a floor value of 6.0 years for all children under six years (1-6 year group: n=18, 24.3% of the cohort), rendering these estimates clinically unreliable, a critical limitation. The 16-Bit Physis AI software platform achieved the lowest AI-related MAE across all three age groups (1-6 yrs: 1.07; 7-12 yrs: 1.39; 13-18 yrs: 1.25).

**Table 6:** Mean Absolute Error (MAE) of manual Greulich and Pyle (GP) assessment and the three AI-based bone age assessment platforms stratified by age group. Lower MAE values indicate a smaller difference from chronological age. Values are presented in years.

Age group	n	GP atlas	Xrayhead	16-Bit Physis	FreeBoneAge
1-6 years	18	0.47	3.71	1.07	1.47
7-12 years	17	1.10	2.71	1.39	1.39
13-18 years	39	0.74	1.30	1.25	1.84

**Figure 7:**
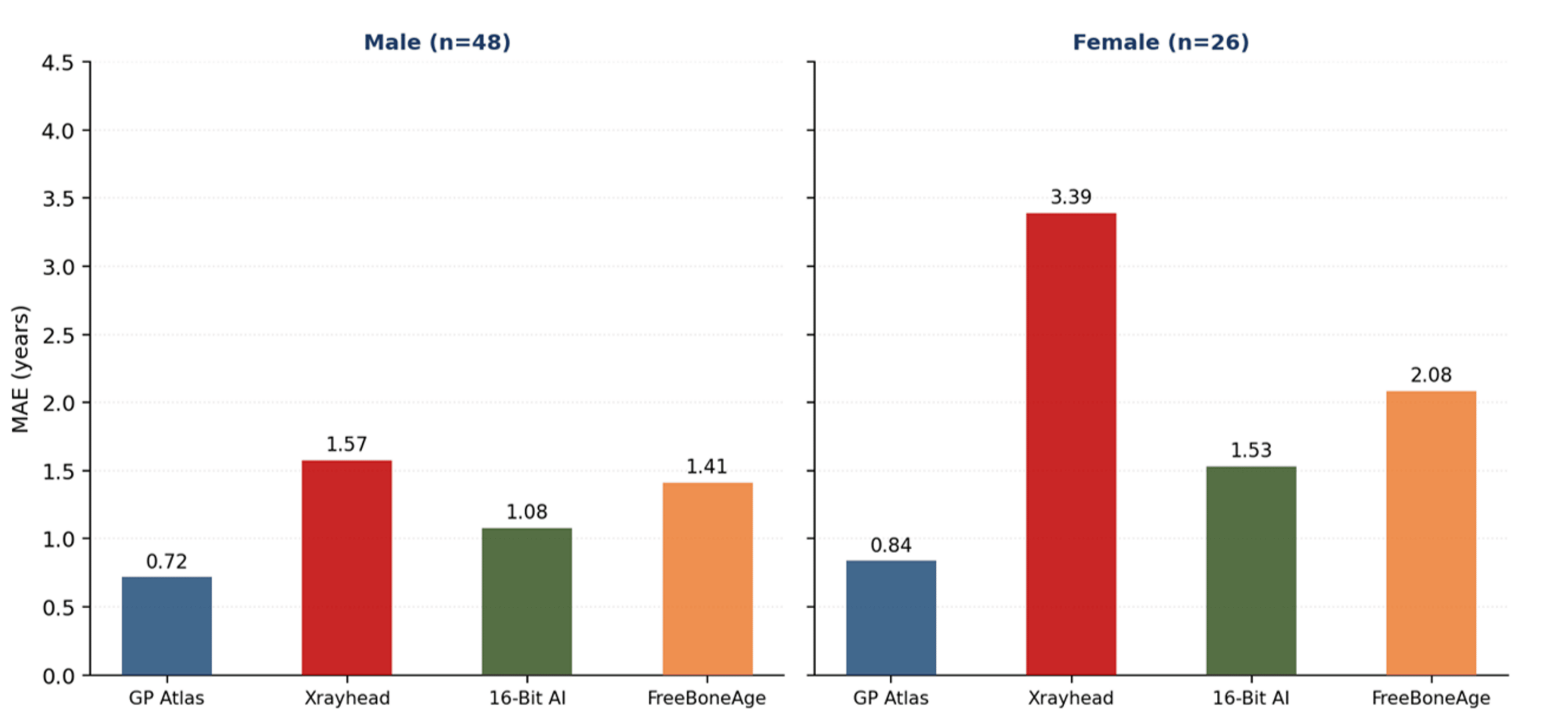
Sex-stratified comparison of bone age assessment accuracy across evaluated methods with male participants (n = 48) and female participants (n = 26). The graph compares Mean Absolute Error (MAE) according to sex for the Greulich and Pyle (GP) atlas, Xrayhead, 16-Bit Physis, and FreeBoneAge. Lower MAE values indicate greater agreement with chronological age and higher accuracy. Xrayhead demonstrated higher error in female participants compared with male participants, suggesting possible sex-related variation in algorithm performance. In contrast, 16-Bit Physis maintained relatively consistent performance across both sexes with comparatively lower error values. All values are presented in years.

Figure 7 shows sex stratified MAE: Xrayhead performed worse in females (MAE 3.39 years) versus males (1.57 years), suggesting a possible trend toward sex-related variation; however, this finding should be interpreted cautiously because of the relatively small female subgroup. The 16-Bit Physis maintained acceptable performance in both sexes (male 1.08; female 1.53 years).

## Discussion

Bone age estimation is central to pediatric endocrinology, growth assessment, and evaluation of pubertal disorders [1]. The GP atlas is the most widely used and available method for skeletal age assessment worldwide and continues to serve as the benchmark against which newer automated methods are evaluated [2]; however, it is subjective and dependent on reader expertise. In high-volume pediatric centres, manual GP assessment can be time-consuming and susceptible to inter- and intra-observer variability. AI-based bone age assessment platforms have emerged as a potential tool to improve efficiency, reproducibility, and workflow standardization [3].

In the present study, we compared the AI platforms Xrayhead [4], 16-Bit Physis [5], and FreeBoneAge [6] with the manual GP atlas method in an Indian pediatric population. All evaluated platforms demonstrated significant correlation with GP assessment; however, substantial differences were observed in agreement, accuracy, and applicability across age groups and sexes.

Among the three evaluated AI platforms, 16-Bit Physis demonstrated the strongest agreement with manual GP assessment. It showed the highest correlation with GP readings, the narrowest limits of agreement, and the lowest AI-related MAE. Importantly, its performance remained consistent across all age groups and both sexes, without apparent age-related restrictions. The small observed bias indicates minimal systematic deviation from GP assessment. The MAE observed in our cohort was comparable to values reported for high-performing AI-based bone age systems in previous studies, although the slightly higher error may reflect population-specific differences in skeletal maturation or variations in algorithm training datasets [3]. Similar findings have been reported by Minty et al., who demonstrated good agreement between automated bone age assessment and manual GP evaluation, supporting the potential role of AI systems as adjuncts to routine clinical practice [7].

FreeBoneAge demonstrated a strong correlation with GP assessment and negligible overall bias, indicating good agreement at the population level. However, its wider limits of agreement suggest greater variability in individual patient assessments when compared with 16-Bit Physis. Reduced performance was observed in younger children and female subjects, highlighting the importance of evaluating subgroup-specific behavior in AI algorithms. These findings are consistent with observations by Alaimo et al., who reported performance differences between AI-based bone age assessment systems despite overall strong correlations with conventional methods [8]. The findings of the present study suggest that while FreeBoneAge may serve as a useful adjunctive tool, caution should be exercised when interpreting individual patient results, particularly in younger age groups.

Among the evaluated systems, Xrayhead demonstrated the weakest overall performance, showing the lowest correlation with GP assessment (r = 0.75) and the widest limits of agreement. A particularly important finding was its inability to assess children younger than six years, producing a fixed output of 6.0 years irrespective of actual skeletal maturity. This limitation affected nearly one-quarter of the study cohort and substantially reduced its clinical utility in younger children. In addition, Xrayhead demonstrated poorer performance in females than in males, suggesting potential sex-related variability in algorithm performance. These findings align with Pape et al., who demonstrated that different bone age algorithms are not interchangeable and may exhibit significant differences in agreement and reliability despite addressing the same clinical task [9].

Collectively, our findings reinforce the growing evidence that AI-based bone age assessment systems can provide rapid and reproducible estimates of skeletal maturity. However, performance varies substantially among available platforms, emphasizing the need for independent validation before widespread clinical implementation. This is particularly relevant in populations that may be underrepresented in the datasets used for algorithm development and training [8,9].

The strengths of this study include the direct comparison of three AI-based bone age assessment platforms using multiple statistical measures as well as subgroup analyses based on age and sex in an Indian pediatric population. However, the limitations should be acknowledged. Given the retrospective design, relatively small sample size, and sex imbalance, these findings should be considered hypothesis-generating and require confirmation in larger prospective multicenter studies. The study was conducted at a single center, which may limit generalizability. Manual GP assessment was performed by a single reader, and neither interobserver nor intraobserver variability was assessed. Future studies incorporating multiple readers and repeat assessments would further strengthen the reference standard.

## Conclusions

Among the three evaluated AI-based bone age assessment platforms, 16-Bit Physis demonstrated the strongest agreement with the manual Greulich and Pyle atlas, the narrowest limits of agreement, and the smallest difference from chronological age among the evaluated AI platforms. FreeBoneAge also showed good agreement but greater individual variability, whereas Xrayhead demonstrated important limitations, particularly in children younger than six years. Although these findings support the potential role of 16-Bit Physis as an adjunctive tool in clinical practice, they should be interpreted in the context of the retrospective single-center design, relatively small convenience sample, and single-reader manual reference standard. Larger prospective multicenter studies are required before widespread clinical implementation can be recommended.
